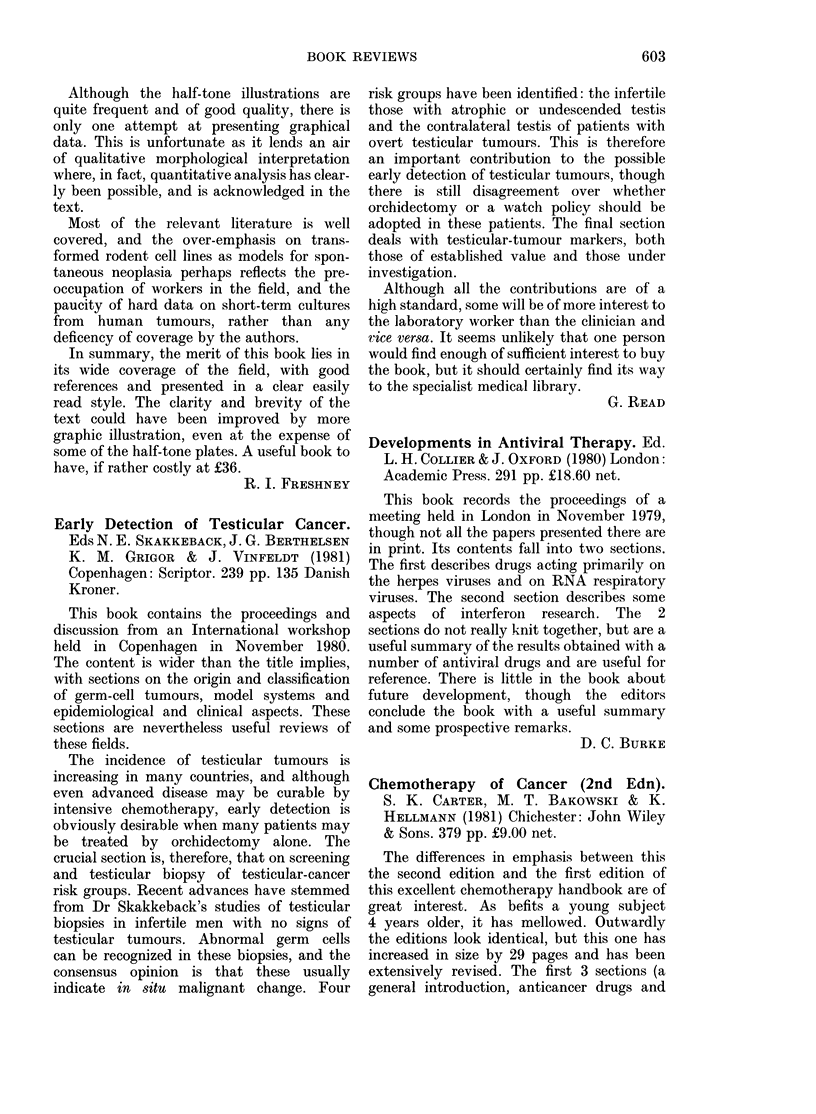# Developments in Antiviral Therapy

**Published:** 1981-10

**Authors:** D. C. Burke


**Developments in Antiviral Therapy.** Ed. L. H. Collier & J. Oxford (1980) London: Academic Press. 291 pp. £18.60 net.

This book records the proceedings of a meeting held in London in November 1979, though not all the papers presented there are in print. Its contents fall into two sections. The first describes drugs acting primarily on the herpes viruses and on RNA respiratory viruses. The second section describes some aspects of interferon research. The 2 sections do not really knit together, but are a useful summary of the results obtained with a number of antiviral drugs and are useful for reference. There is little in the book about future development, though the editors conclude the book with a useful summary and some prospective remarks.

D. C. Burke